# Supplementary material for: Dynamics of infection-elicited SARS-CoV-2 antibodies in children over time
Source: medRxiv. 2022 Jan 25:2022.01.14.22269235. Preprint. [Version 1] doi: 10.1101/2022.01.14.22269235 (PMC8811949; doi:10.1101/2022.01.14.22269235)
Supplement: 1 [file NIHPP2022.01.14.22269235V1-supplement-1.pdf]

medRxiv preprint doi: https://doi.org/10.1101/2022.01.14.22269235; this version posted January 25, 2022. The copyright holder for this preprint (which was not certified by peer review) is the author/funder, who has granted medRxiv a license to display the preprint in perpetuity.
It is made available under a CC-BY 4.0 International license .

Supplemental figures:

Supplemental Table 1. Evidence of SARS-CoV-2 infection among patients without a confirmed SARS-CoV-2 RT-PCR.

Patient ID	Evidence of SARS-CoV-2 infection	Epi-week of household RT-PCR test	Epi-week of participant symptom onset
C15	Experienced syptomatic infection, developed MIS-C, neutralization and nucleocapsid antibodies confirmed through serological testing; this child is listed un the MIS-C subset in inclusion flowchart.	not applicable	2020 week 11 - acute<br>2020 week 18 - MIS-C
C12	Known PCR-positive household infection (family member with long COVID who was not tested until well after initial household outbreak), entire family experienced symptoms consistent with SARS-CoV-2 infection, neutralization and nucleocapsid antibodies confirmed through serological testing	2020 week 20	2020 week 11
C20	Known PCR-positive household infection, experienced symptoms consistent with SARS-CoV-2 infection, neutralization and nucleocapsid antibodies confirmed through serological testing	unknown	2020 week 12
C23	Known PCR-positive household infection, experienced symptoms consistent with SARS-CoV-2 infection, neutralization and nucleocapsid antibodies confirmed through serological testing	two family members positive both in 2020 week 49	2020 week 49
C14	Known PCR-positive contacts, experienced symptoms consistent with SARS-CoV-2 infection, neutralization and nucleocapsid antibodies confirmed through serological testing	2020 week 48	2020 week 48

medRxiv preprint doi: https://doi.org/10.1101/2022.01.14.22269235; this version posted January 25, 2022. The copyright holder for this preprint (which was not certified by peer review) is the author/funder, who has granted medRxiv a license to display the preprint in perpetuity.
It is made available under a CC-BY 4.0 International license .

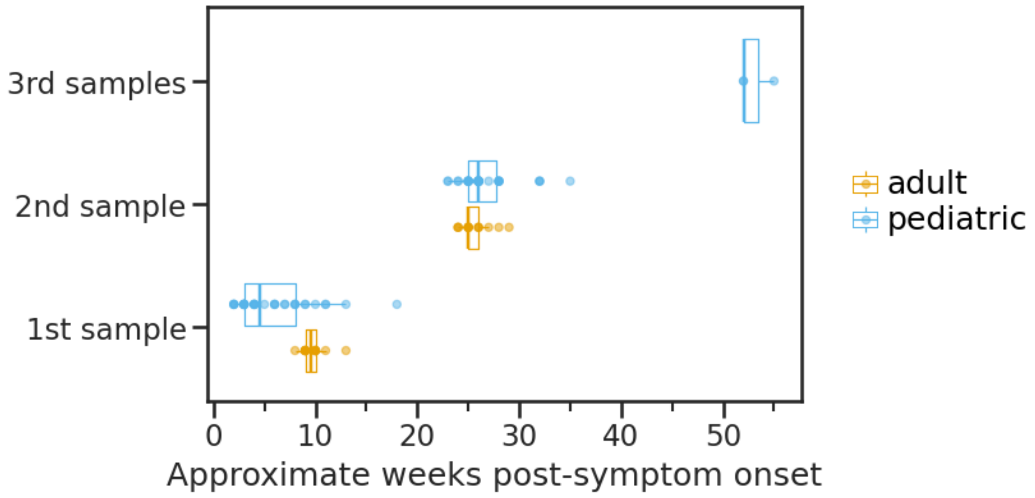

Supplemental figure 1. Distribution of specimen collections in children and adults.

A

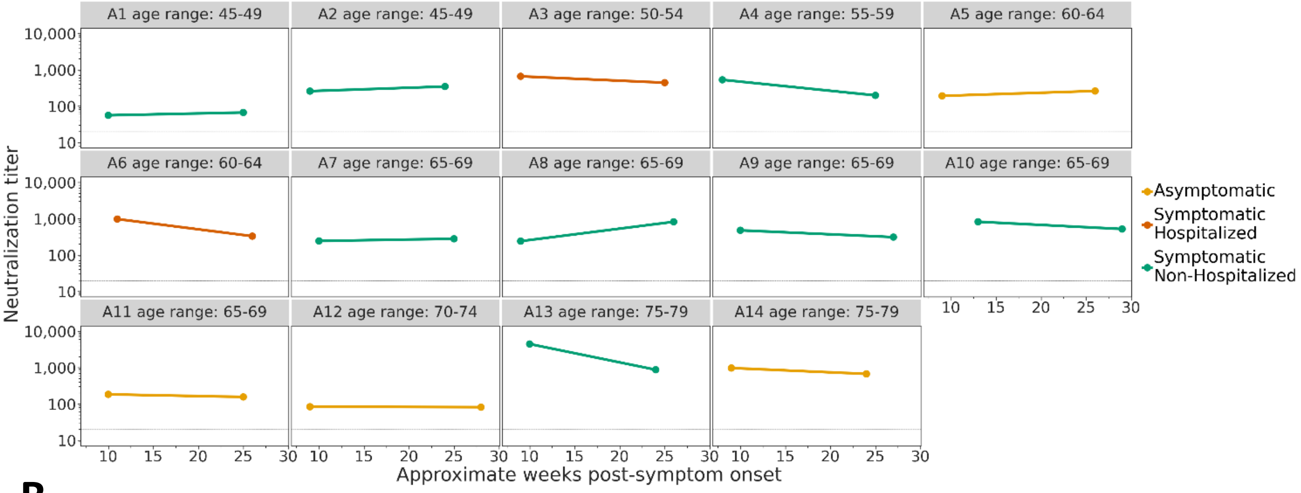

B

medRxiv preprint doi: https://doi.org/10.1101/2022.01.14.22269235; this version posted January 25, 2022. The copyright holder for this preprint (which was not certified by peer review) is the author/funder, who has granted medRxiv a license to display the preprint in perpetuity.
It is made available under a CC-BY 4.0 International license .

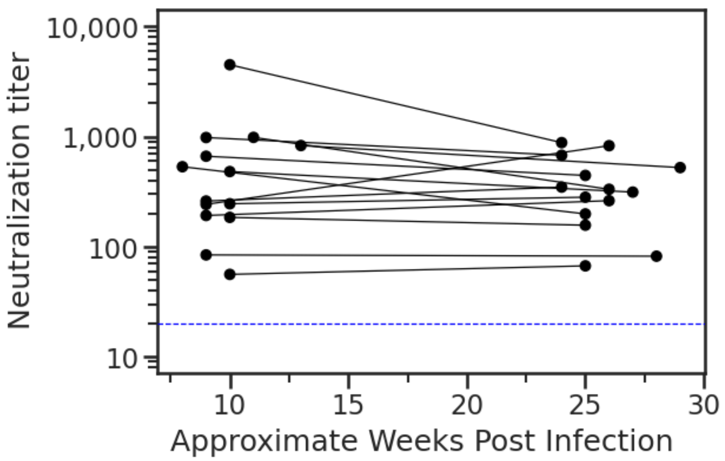

Supplemental figure 2. *Neutralization titers in adults over time.* **A**) Neutralizing antibody titers in 14 adults with confirmed SARS-CoV-2 infection followed prospectively over time shown as weeks post-symptom onset, x axis. **B**) Aggregated neutralization titers for all adults. Dotted horizontal lines indicate the limit of detection (20).

medRxiv preprint doi: https://doi.org/10.1101/2022.01.14.22269235; this version posted January 25, 2022. The copyright holder for this preprint (which was not certified by peer review) is the author/funder, who has granted medRxiv a license to display the preprint in perpetuity.
It is made available under a CC-BY 4.0 International license .

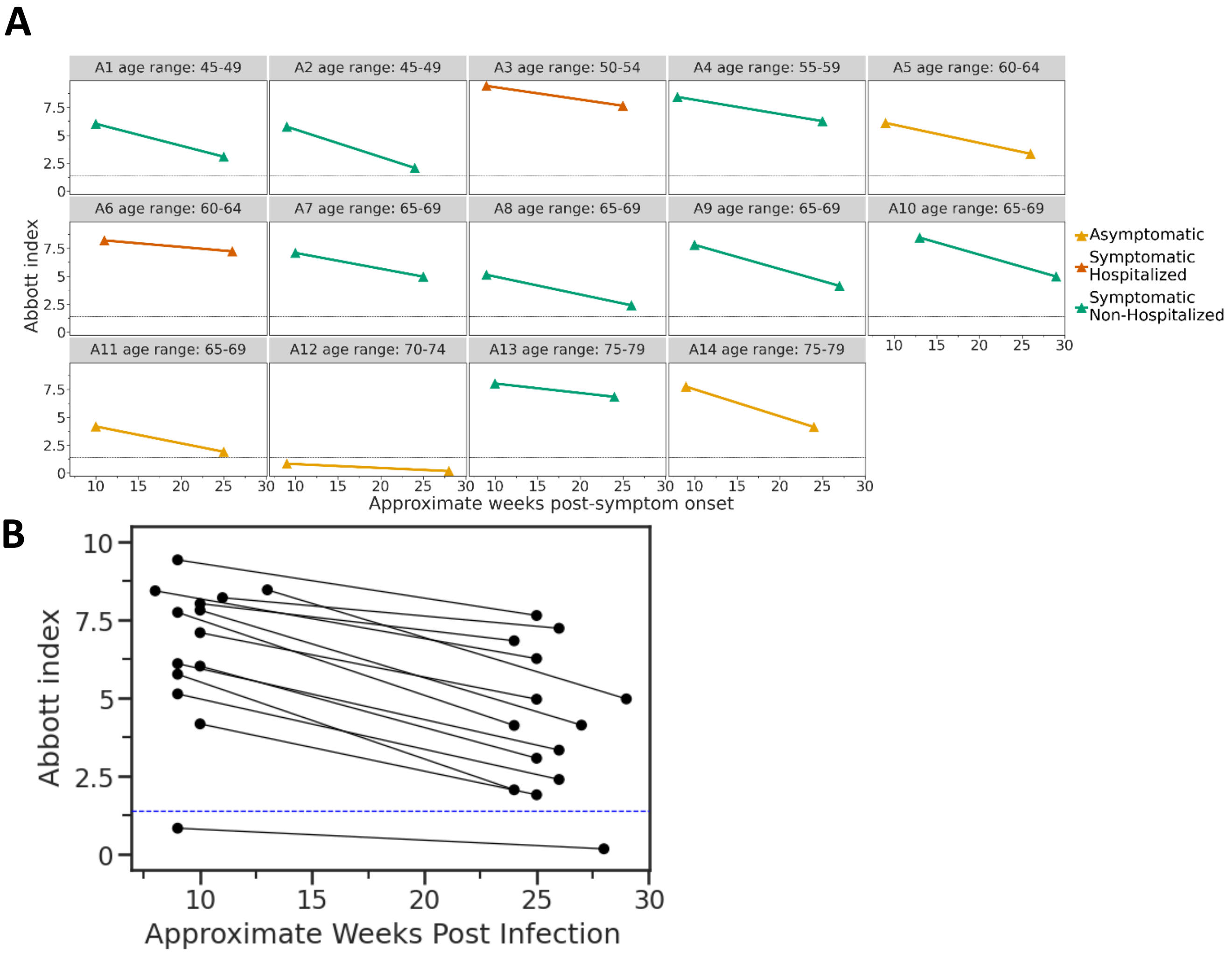

Supplemental figure 3. *Nucleocapsid-binding antibody levels in adults over time.* **A**) The SARS-CoV-2 IgG assay was used to determine SARS-CoV-2 nucleocapsid-binding antibody in 14 adults followed prospectively over time shown as weeks post-symptom onset, x axis. **B**) Aggregated index values for all adults. Dotted horizontal lines indicate the limit of detection for the SARS-CoV-2 IgG assay (1.40).

Supplemental Table 2. *Naming of adults across publications.*

Naming in Crawford et al. 2020 (3)	Naming in the present study

medRxiv preprint doi: https://doi.org/10.1101/2022.01.14.22269235; this version posted January 25, 2022. The copyright holder for this preprint (which was not certified by peer review) is the author/funder, who has granted medRxiv a license to display the preprint in perpetuity.
It is made available under a CC-BY 4.0 International license .

PID 13	A3
PID 3C	A1
PID 4C	A2
PID 6C	A6
PID 7C	A7
PID 11C	A4
PID 12C	A10
PID 22C	A9
PID 23C	A8
PID 24C	A13
PID 103C	A12
PID 113C	A14
PID 117C	A11
PID 200C	A5
